# From chemically defined hiPSCs to self-organizing cardiac organoids: current strategies guided by developmental signaling

**DOI:** 10.1186/s13287-026-04996-5

**Published:** 2026-04-10

**Authors:** Hao Yang, Yunqian Zeng, Yiyang Teng, Qi Zhao, Minbo Hou, Zhikai Cheng, Xijie Wang

**Affiliations:** 1 School of Pharmaceutical Sciences, Fudan University, Shanghai, 201203 China; 2 Shanghai InnoStar Bio-tech Co., Ltd, Shanghai, 201203 China

**Keywords:** Cardiac organoids, hiPSCs, Chemically defined culture, Self-organized differentiation, Wnt signaling pathway, Drug-induced cardiotoxicity assessment

## Abstract

Cardiovascular research and drug development remain constrained by the limited translational relevance of conventional animal and in vitro cellular models, which represent a major bottleneck in the field. In recent years, self-organizing cardiac organoids derived from hiPSCs have emerged as a promising alternative. These organoids recapitulate key aspects of human cardiac development, physiological function, and disease-related features in vitro, thereby providing a powerful platform for mechanistic studies and drug screening. The successful establishment of such systems relies on two critical components. First, chemically defined and xeno-free hiPSC culture systems are essential for ensuring experimental standardization and reproducibility. Second, a comprehensive understanding of key developmental signaling pathways (e.g., Wnt and BMP/Activin) and their precise spatiotemporal regulation is required to enhance the maturation and biomimetic fidelity of three-dimensional cardiac models. In this review, we summarize the progression from standardized hiPSC culture systems to the generation of self-organizing cardiac organoids, with a particular focus on the regulatory mechanisms and engineering strategies underlying core developmental signaling pathways. We further discuss the potential applications of this technology in precision cardiovascular medicine.

## Background

Cardiovascular disease (CVD) remains a leading global health challenge and was responsible for approximately 19.41 million deaths in 2021, highlighting the urgent need for improved research strategies [1]. Human induced pluripotent stem cell (hiPSC) technologies and animal models have been widely applied in cardiovascular research and have played indispensable roles in advancing the field [2, 3]. However, these models exhibit inherent limitations. Specifically, two-dimensional (2D) culture systems lack three-dimensional (3D) cell–extracellular matrix (ECM) interactions and fail to recapitulate the structural complexity of native cardiac tissue [4, 5]. In addition, animal models often cannot accurately reproduce human-specific electrophysiological characteristics due to interspecies differences [6, 7]. These limitations significantly hinder the investigation of cardiac disease mechanisms and reduce the predictive power of preclinical drug development [8]. The emergence of cardiac organoids offers a new paradigm for overcoming the aforementioned bottlenecks. These organoids are typically derived from human embryonic stem cells (hESCs) and hiPSCs, and self-assemble under specific inductive conditions into 3D structures containing multiple cardiac cell types, capable of recapitulating certain functions and characteristics of the in vivo heart [9]. Among these, hiPSC-based self-assembling cardiac organoid technology, by recapitulating early heart developmental processes in vitro, has enabled the initial construction of physiologically relevant cardiac models [10, 11]. Currently, this technology is increasingly being applied in various fields, including disease modeling, drug toxicity assessment, and studies on cardiac embryonic development mechanisms [12, 13]. However, the widespread application of this technology depends on the reliability of the cell source and the reproducibility of the models. This requires maintaining the stable expansion and directed differentiation of hiPSCs in culture systems with chemically defined, xeno-free components [14]. Although several chemically defined hiPSC media have been successfully developed, the accompanying xeno-free adhesion substrate systems still require further optimization [15, 16]. Beyond culture conditions, the organoid construction strategy also determines its physiological relevance. Constructing self-organizing cardiac organoids from hiPSCs necessitates precise spatiotemporal regulation of key developmental signaling pathways, which requires an in-depth understanding of signaling dynamics and assembly mechanisms [17]. Of note, the development of such models aligns closely with the 3Rs principle (Replacement, Reduction, and Refinement) and is garnering increasing policy support. For instance, the U.S. FDA Modernization Act 2.0 enables the use of “novel methodology” models, including cardiac organoids, as alternatives to animal testing in certain contexts [18, 19].

Based on the progress and challenges outlined above, this review summarizes recent advances in xeno-free hiPSC culture systems and strategies for constructing self-organizing cardiac organoids. It outlines their differentiation logic and applications in cardiac disease modeling, aiming to provide a reference for the development of cardiovascular disease models and translational research.

## Establishment and regulation of hiPSC culture systems

Between 2006 and 2007, Takahashi and Yamanaka’s team successfully reprogrammed somatic cells into iPSCs using four transcription factors: Oct4, Sox2, Klf4, and c-Myc (collectively known as OSKM) [20, 21]. The resulting iPSCs closely resemble ESCs in morphology, self-renewal capacity, and pluripotency, making them a revolutionary tool for organoid research [22]. The maintenance of hiPSC pluripotency relies on an intricate network of intrinsic and extrinsic regulatory signals. At its core is an endogenous gene network centered on the OSKM factors: Oct4 and Sox2, in cooperation with Nanog, maintain epigenetic homeostasis and self-renewal [23, 24]; Klf4 cooperates with Oct4 to activate the pluripotency network, while c-Myc primarily promotes cell cycle progression and proliferation [25]. This intrinsic network is complemented by precise regulation from exogenous signaling pathways. For instance, the FGF pathway (particularly FGF2) maintains cells in a primed state of pluripotency by activating ERK1/2; the Insulin/IGF pathway regulates diverse cellular processes via the AKT signaling hub; and the TGF-β/Nodal/Activin pathway, primarily through Activin A, drives the expression of the core pluripotency network. These pathways collectively sustain the hiPSC state [26–30].

**Fig. 1:**
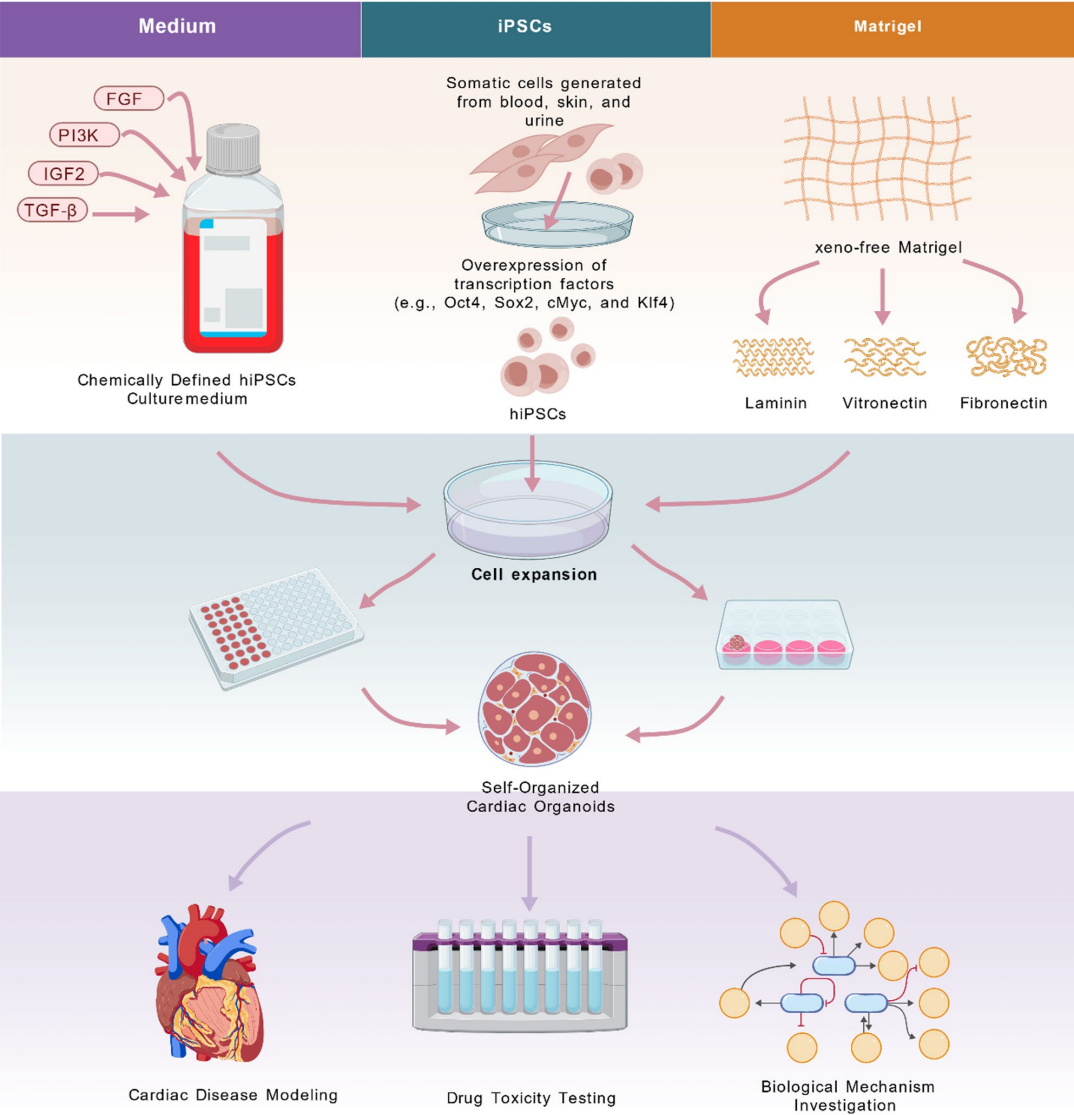
Generation of hiPSCs and culture for self-organizing cardiac organoids: Human somatic cells are reprogrammed to hiPSCs via OSKM factors. hiPSCs are maintained in a chemically defined medium utilizing key signaling pathways (FGF, PI3K/AKT, IGF, TGF-β) and a xeno-free extracellular matrix (e.g., Laminin, Vitronectin). Through self-organization, iPSCs differentiate into cardiac organoids, which are applicable for disease modeling, drug toxicity testing, and mechanistic studies

The unique ability of hiPSCs to retain donor-specific genetic backgrounds makes them an ideal model for in vitro studies of human cardiac biology. Their culture systems have undergone a paradigm shift, transitioning from xenogeneic-containing systems toward chemically defined, xeno-free alternatives [31]. This evolution has significantly reduced biological variability while enhancing experimental standardization and reproducibility. It is worth emphasizing that culture conditions substantially impact the efficiency of hiPSC differentiation into cardiomyocytes, as well as their electrophysiological properties and maturation state [32, 33]. Therefore, the adoption of xeno-free culture systems is critical for the precise control of cardiac differentiation. These systems reduce batch-to-batch variations inherent to xenogeneic components and minimize the risk of unintended immune responses and functional interference [34, 35]. In summary, the continuous refinement of hiPSC culture systems is a fundamental prerequisite for generating high-quality, reproducible cardiac organoids. The process from human somatic cells to functional cardiac organoids and their applications is illustrated in Fig. 1 [36].

### Development of chemically defined media

The field of hiPSC culture has shifted toward chemically defined media to overcome the significant batch-to-batch variability associated with the undefined composition of traditional serum-containing systems [37]. Early commercial media, such as mTeSR [38] and StemPro [39], supported stable hiPSC proliferation and gained widespread use. However, they often contained xenogeneic components like BSA and were costly [40], which drove the need for further optimization. A major step forward was the E8 medium, which was designed by rigorously screening components to eliminate albumin entirely. Its formulation, comprising only eight essential constituents (including insulin, transferrin, FGF2, and TGF-β1), effectively maintains hiPSC pluripotency while significantly streamlining the medium composition [41]. More recently, Kuo et al.developed the B8 medium through systematic optimization of medium constituents. The formulation contains eight core components—including insulin, transferrin, ascorbic acid 2-phosphate, sodium selenite, a thermostable FGF2 variant (FGF2-G3), TGFβ3, NRG1, and sodium bicarbonate—in DMEM/F12 basal medium. This system supported the long-term stable culture of 34 different hiPSC lines and maintains genomic stability and high pluripotency while being compatible with a “weekend-free” feeding protocol. This advance substantially reduces both culture complexity and cost [42]. Hua et al. developed the ON2/AscleStem medium, which employs a uniquely reconstructed growth factor network. This formulation replaces TGF-β with Activin A, reduces FGF2 concentration, and supplements Leukaemia Inhibitory Factor to support pluripotency-related signaling. This medium demonstrates broad substrate compatibility and excellent long-term culture stability [43]. Another innovative strategy integrates advanced engineering technologies with chemical culture supplements. One representative method combines low-pressure microfluidic sorting with the multifunctional CEPT cocktail (containing chroman 1, emricasan, polyamines, and trans-ISRIB). This system uses a low-shear-stress mechanism for precise single-cell dispensing, while the CEPT reagents modulate multiple cellular stress response pathways. It achieves a high clonal formation within 7–14 days [44]. Collectively, these advancements reflect a profound paradigm shift in hiPSC research—from empirical practices toward a precisely controlled and engineered approach.

### Compositional definition of ECM

In parallel with the evolution of culture media, the ECM, another critical element in hiPSC culture, has transitioned from biologically derived extracts toward chemically defined materials. Early hiPSC culture depended on feeder layers of γ-irradiated mouse embryonic fibroblasts (MEFs). However, irradiation irreversibly alters the secretory profile of these cells. Combined with their inherent species-specific variations, this results in significant heterogeneity in growth factor secretion and ECM deposition, severely compromising experimental reproducibility [45]. To address these limitations, feeder-free systems were developed. Matrigel, extracted from mouse Engelbreth-Holm-Swarm (EHS) sarcoma, was the first widely adopted solution. Although its 3D hydrogel network provides biomimetic support, its tumor origin entails a triple drawback: potential pathogen risks, substantial batch-to-batch variation, and the presence of uncontrollable endogenous growth factors, limiting its use in precision research [46, 47].

These shortcomings motivated the field to pursue precise reconfiguration using recombinant matrix proteins. Laminin heterotrimers, as core components of the basement membrane, became a major focus [48, 49]. Laminin-511 (LN-511) was shown to maintain cell-cell adhesion via the E-cadherin/PI3K pathway while inhibiting apoptosis through α6β1 integrin-mediated activation of the Fyn-RhoA-ROCK cascade, thereby collectively sustaining pluripotency [50]. Moreover, both LN-511 and its homolog LN-521 efficiently support hiPSC expansion and pluripotency maintenance [51, 52]. Yap et al. confirmed that LN-221, a core component of the cardiac basement membrane, not only efficiently induced cardiomyocyte differentiation from hiPSC but also exhibited excellent batch-to-batch stability. Their GMP-produced recombinant human LN-221, when combined with LN-521, was shown to synergistically activate the Wnt pathway, inducing a transcriptomic signature favorable for cardiac differentiation [53]. Beyond laminins, vitronectin has also been validated as an effective Matrigel substitute [54–56]. This glycoprotein primarily mediates cell adhesion via integrin αVβ5. Its truncated form, VTN-N, when used in E8 medium with appropriate single-cell support conditions, can efficiently support hiPSC attachment and single-cell culture [41]. Liu et al. developed a mixed strategy using recombinant vitronectin and LN-511, thereby enabling stable hiPSC expansion on uncoated dishes. The expanded cells maintained pluripotency and demonstrated efficient differentiation potential into all three germ layers and cardiomyocytes [57]. Fibronectin is critical for cardiac-directed differentiation. Early work by Baxter et al. showed that a fibronectin coating alone is sufficient to maintain hiPSC in an undifferentiated state long-term in defined media, with adhesion depending on the synergistic action of integrin β1 and α5 subunits [58]. Zhang et al. revealed the differential regulation of cardiomyogenesis by ECM proteins: fibronectin significantly promoted it, whereas LN-111, LN-521, and collagen IV are inhibitory. The study confirmed that the initiation of cardiac differentiation strictly depends on the functional presence of fibronectin in the ECM, an effect potentially mediated by the integrin integrin α4β1 and αvβ1 complex activating the ILK pathway and upregulating pAKT levels [59]. Building on this, Kupfer et al. developed a defined, biomimetic ECM bio-ink composed of fibronectin, LN-111, and collagen methacrylate, which effectively promotes hiPSC cardiac differentiation [60].

The ultimate strategy in chemical definition involves fully synthetic substrate materials. Nasir et al. established that a nanoscale, phase-separated blend of poly (tricyclodecane-dimethanol diacrylate) and poly (butyl acrylate) (2:1 v/v)—discovered via high-throughput screening of over 600 polymers in E8 medium—enables long-term human pluripotent stem cell (hPSC) expansion on standard surfaces [61]. Cho et al. developed a novel synthetic polymer film, pGC2, which supports stable hPSC expansion for over 10 passages, while maintaining pluripotency marker expression and a normal karyotype [62]. In summary, the evolution of ECMs—from animal-derived Matrigel to precisely defined recombinant proteins and further to fully synthetic polymers—clearly progresses toward the goals of defined composition, controlled functionality, and batch stability. These fully defined ECM systems fundamentally circumvent the risks of xenogeneic components. Their modular nature permits optimization for specific differentiation lineages, establishing a solid foundation for standardizing hiPSC downstream applications and their clinical translation.

## Construction strategies for hiPSC-based cardiac organoids and developmental signaling

The establishment of standardized hiPSC culture systems has laid the groundwork for generating highly reproducible cardiac organoids. Current strategies for constructing hiPSC-based cardiac organoids primarily revolve around three major technological approaches: engineered cardiac organoids, organ-on-a-chip platforms, and self-organizing cardiac organoids [63]. Among these, self-organizing cardiac organoids have garnered particular attention due to their ability to closely recapitulate human heart development through the embryoid body-based differentiation pathway. These models leverage the intrinsic self-assembly capacity of hiPSCs to spontaneously form complex 3D structures comprising multiple cardiac lineage cell types, ultimately exhibiting morphological and functional characteristics similar to those of the native heart [64–66]. Although mouse models have been extensively studied, interspecies differences limit the direct extrapolation of findings from these models to human physiology [67, 68]. The successful construction of such models must be grounded in a deep understanding of human cardiac development. The human heart, being the first functional organ formed in the embryo, initiates its development during gastrulation [69]. This process can be broadly divided into early morphogenesis and later maturation stages, with in vitro modeling primarily aiming to recapitulate the former. Critically, even before recognizable cardiac structures emerge, the embryo precisely regulates the differentiation and migration of cardiac precursor cells [70]. The spatiotemporal precision of this process depends on the correct establishment of the embryonic axial pattern: following gastrulation, cardiac progenitors derived from the mesoderm, through a series of morphogenetic events (including cell specification, directed differentiation, and migration), progressively construct the embryonic heart with primitive chamber structures [71].

This process is driven by the coordinated development of two cardiac fields: the First Heart Field (FHF) primarily contributes to the left ventricle and parts of the atria, whereas the Second Heart Field (SHF) gives rise to the right ventricle, outflow tract, and most of the atria [12]. However, the mature heart comprises four functionally specialized chambers, which differ not only in morphology and function but also exhibit significant variations in cellular composition, origin, and proportion. For instance, in atrial tissue, cardiomyocytes account for approximately 30.1% of cells, with the microenvironment consisting of fibroblasts, mural cells, endothelial cells, and immune cells. In contrast, ventricular regions (including the apex and interventricular septum) are predominantly composed of ventricular cardiomyocytes, accompanied by higher proportions of mural cells and fibroblasts, while the proportions of endothelial cells and immune cells are relatively lower [72]. This highly specialized cellular composition and spatial distribution impose stringent requirements on in vitro cardiac organoids—they must accurately mimic the core signaling networks and cell-cell interactions that drive cardiac development [73, 74]. Therefore, a thorough decoding of the molecular mechanisms governing heart development is a fundamental prerequisite for successfully building physiologically relevant self-organizing cardiac organoids.

**Fig. 2:**
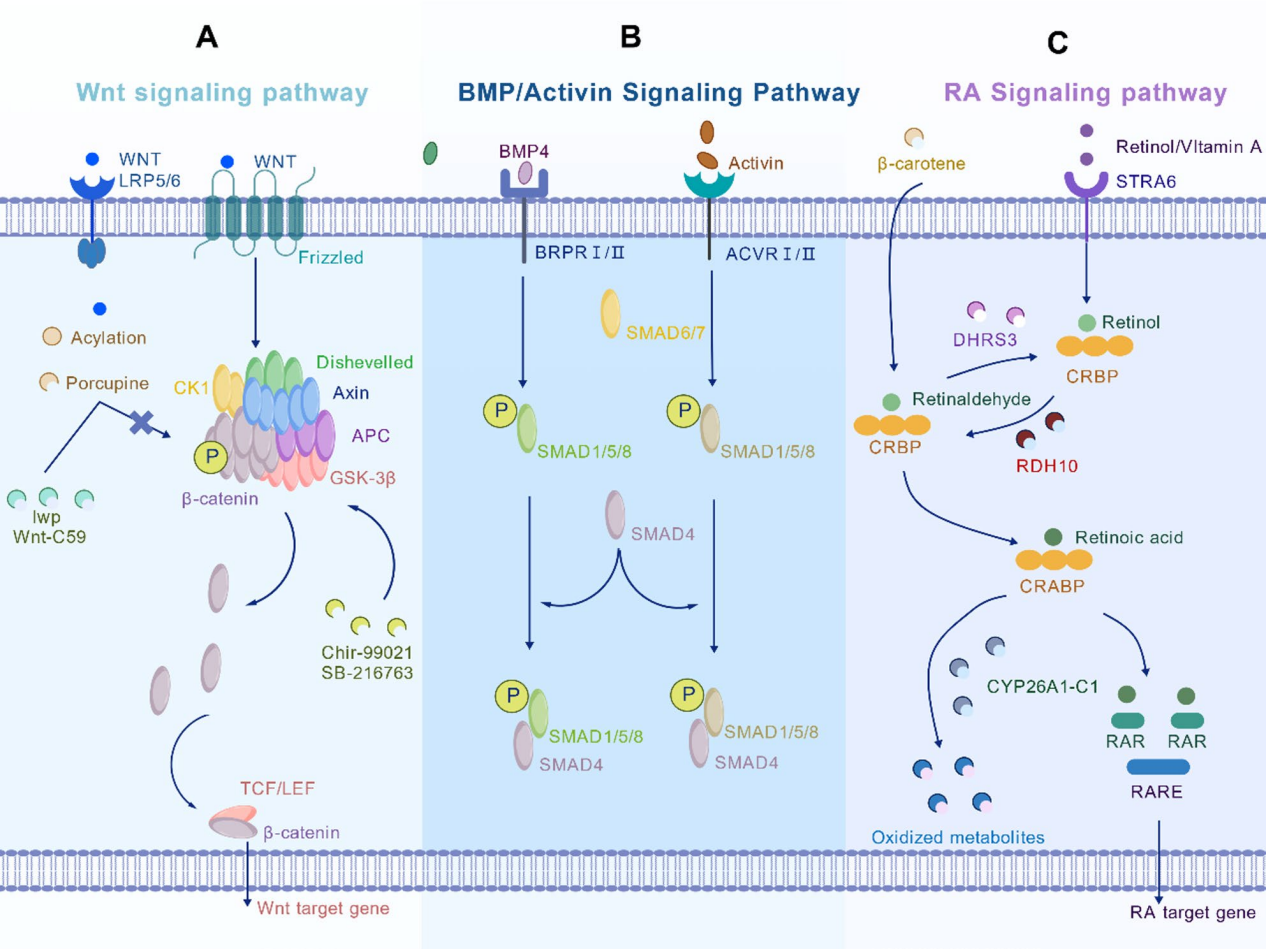
Core signaling pathways governing early cardiac development in organoids. **A** Wnt/β-catenin pathway is dynamically regulated through sequential activation and inhibition to stage-specifically direct cardiac differentiation; **B** BMP/SMAD and Activin/SMAD pathways are transduced through distinct SMAD proteins (SMAD1/5/8 and SMAD2/3, respectively) and form an integrated network; **C** RA metabolism involves synthesis from retinol, with cellular levels balanced by nuclear signaling via RAR/RXR and degradation by CYP26 enzymes

### Dynamic regulation of the Wnt signaling pathway

The Wnt/β-catenin signaling pathway orchestrates early cardiac development through its dynamic activation and inhibition. During embryonic gastrulation, canonical Wnt signaling activation is crucial for the expansion and maintenance of mesoderm-derived cardiac progenitors. Following Porcupine (Porcn)-mediated acylation, secreted Wnt ligands bind to the Frizzled (Fzd) receptor and its LRP5/6 co-receptors. This complex recruits and activates the cytoplasmic protein Dishevelled (Dvl), which in turn interacts with the β-catenin destruction complex (composed of Axin, GSK-3β, and APC). Wnt signaling prevents β-catenin ubiquitination and degradation by this complex. The stabilized β-catenin subsequently translocates into the nucleus, where it associates with TCF/LEF transcription factors to initiate the transcriptional program of downstream cardiac precursor genes [75].

A hallmark of Wnt signaling is its characteristic biphasic activity pattern during heart development, which can be recapitulated in vitro using small molecules. CHIR99021, a potent and selective GSK-3β inhibitor, effectively activates the Wnt pathway by stabilizing β-catenin and is a standard tool for inducing mesoderm formation in hiPSC cardiac differentiation protocols [76]. Following this initial activation, Wnt pathway inhibition is essential to drive the differentiation of cardiac progenitors into functional cardiomyocytes [77]. The Porcupine protein is critical here, as its palmitoylation of Wnt ligands in the endoplasmic reticulum is a prerequisite for their secretion and activity. Thus, Porcupine inhibitors such as IWP-2 or Wnt-C59 inhibit ligand palmitoylation and secretion, enabling specific pathway inhibition [78].

This tightly regulated, biphasic model has been successfully integrated into hiPSC cardiac organoid differentiation protocols. The initial addition of a Wnt agonist efficiently induces cardiac mesoderm, while subsequent switching to a Porcupine inhibitor within a specific time window promotes cardiac specification and cardiomyocyte differentiation by modulating downstream signaling networks [79–81]. Figure 2A [36] illustrates this core Wnt signaling mechanism governing cardiac organoid differentiation.

### Cooperative regulation by TGF-β superfamily members

The TGF-β superfamily, comprising key developmental signaling proteins such as BMP4 and Activin A, cooperates with the Wnt pathway to precisely regulate the specification and fate determination of cardiac progenitors at critical stages [82]. As detailed in Fig. 2B [36], the molecular mechanisms involve ligand-specific SMAD activation: BMP signaling triggers the phosphorylation of SMAD1/5/8, whereas Activin A signaling phosphorylates SMAD2/3. In both cases, the phosphorylated SMADs form a complex with SMAD4, translocate to the nucleus, and regulate target gene expression [83]. Building on this, the signaling gradient between Activin A and BMP4 further determines atrial versus ventricular lineage fate: a higher BMP4/Activin A ratio promotes ventricular lineage differentiation, while a lower ratio favors atrial lineage formation [84]. On a temporal dimension, BMP4 and Wnt signaling exhibit a dynamic antagonistic relationship. During early differentiation, BMP4 promotes the differentiation of anterior SHF progenitors into ventricular cardiomyocytes by maintaining cTnT expression; inhibition of this pathway reduces the proportion of cardiomyocytes [85]. In contrast, during later differentiation stages, Wnt3a suppresses cardiomyocyte maturation by inhibiting BMP4 expression—an effect that can be reversed by exogenous BMP4 supplementation [79]. Of note, inhibition of SMAD selectively suppresses the FHF marker HCN4 without affecting the SHF marker TBX1 or Wnt activity. This indicates that FHF specification is driven by the BMP/SMAD pathway, whereas SHF specification relies on a non-SMAD-dependent BMP-Wnt signaling cascade [86]. Taken together, BMP4 and Activin A, by balancing SMAD1/5/8 and SMAD2/3 pathway activity in a dose-dependent manner, precisely regulate mesodermal subtype specification, thereby serving as key regulatory nodes for cardiac lineage induction.

### Multidimensional regulation of RA signaling

Retinoic acid (RA) plays multifaceted and pivotal roles in cardiac development, critically regulating the establishment of anteroposterior patterning, epicardial development, and ultimate cardiac morphogenesis [87]. As the most biologically active metabolite of vitamin A, RA is synthesized through a tightly regulated enzymatic cascade. Precursors include retinol (entering via the STRA6 receptor) and retinal (generated from β-carotene oxidation). Intracellularly, retinol dehydrogenase 10 (RDH10) oxidizes retinol to retinal, which is subsequently converted to all-*trans* retinoic acid (ATRA) by ALDH1A/RALDH enzymes. Newly synthesized RA binds to cellular retinoic acid-binding proteins (CRABP1/2) and is then partitioned between two primary fates: nuclear signaling or enzymatic degradation. For canonical nuclear signaling, RA binds to retinoic acid receptor (RAR)/retinoid X receptor (RXR) heterodimers, with CRABP2 reported to facilitate RA delivery to nuclear receptors in some contexts. This complex then recognizes retinoic acid response elements (RAREs) on target genes to initiate transcription. Alternatively, CRABP can direct RA to the CYP26 enzyme system for degradation, a key process maintaining RA homeostasis [88, 89]. The RA biosynthetic and metabolic pathways are depicted in Fig. 2C [36].

Beyond its metabolism, RA signaling precisely regulates cardiac lineage fate through its concentration gradient. Research by Tan et al. demonstrated that during hiPSC differentiation, RA addition at the cardiac progenitor stage specifically induces NR2F1 expression, promoting atrial-like cardiomyocyte differentiation. Conversely, RA signaling inhibition biases cells toward a ventricular lineage [90]. However, proper signaling levels are critical, as excess RA disrupts normal development and can severely impair outflow tract (OFT) formation [91]. Studies in animal models confirm this dose-dependency: RA deficiency results in impaired ventricular trabeculation and defective OFT septation, whereas RA excess impedes OFT ridge development [92].

The synthesis and function of RA involve a complex regulatory network and synergistic interactions with other pathways. Branco et al. reported a stage-dependent, biphasic role for RA in organoid models. When combined with Wnt activation, RA predominantly directs epicardial lineage specification. In the absence of Wnt signaling, it promotes myocardial differentiation while suppressing hepatic fate. Furthermore, combined RA and BMP treatment upregulates cardiac progenitor markers and mesodermal markers, thereby supporting the expansion and specification of the cardiac mesodermal lineage [93]. This requirement for precise combinatorial signaling is further emphasized by Wiesinger et al., who systematically outlined strategies for directing hiPSCs into specific cardiac lineages using RA [87]. For instance, generating sinoatrial node-like cardiomyocytes requires lower concentrations of BMP4 (2.5 ng/mL) and RA (0.25 µM), whereas epicardial cell specification necessitates higher concentrations of BMP4 (10–50 ng/mL) and RA (1–4 µM). This interplay underscores that recapitulating cardiac cellular diversity requires the precise coordination of signaling pathway dosage, timing, and combination.

Collectively, these signaling pathways—including Wnt, BMP/Activin, and retinoic acid—do not function independently but rather form an integrated regulatory network. Their coordinated, stage-specific, and dosage-dependent interactions are essential for guiding cardiac lineage specification and organoid self-organization. Therefore, precise modulation of these pathways represents a central principle in the construction of physiologically relevant cardiac organoids.

### Innovations in cardiac organoid differentiation strategies

**Fig. 3:**
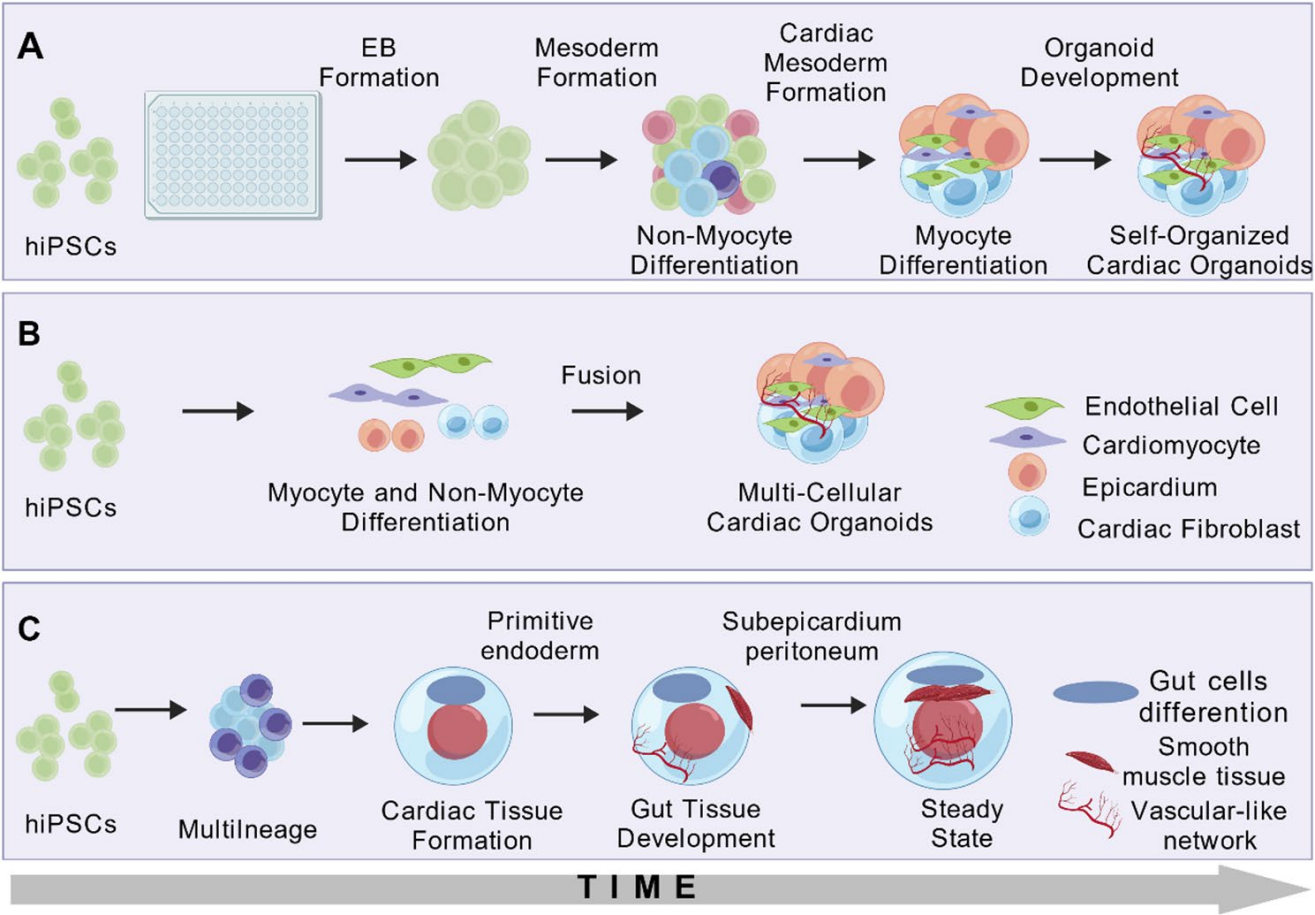
Strategies for Generating 3D Cardiac Organoids. **A.** EB Differentiation: hiPSCs aggregate into EBs and undergo stepwise differentiation in 3D suspension, yielding multi-lineage cardiac organoids; **B.** Lineage-Specific Co-assembly: Pre-differentiated cardiac progenitors are combined to assemble into structured organoids; **C.** Multi-germ Layer Co-development: Co-development of cardiac mesoderm and gut endoderm supports the emergence of post-heart tube stage cardiac features

Leveraging insights into core developmental pathways such as Wnt, BMP/Activin, and RA, researchers have devised diverse protocols to guide hiPSC self-organization into cardiac organoids. These strategies aim to recapitulate embryonic signaling dynamics—notably the sequential activation and inhibition of Wnt—to drive the emergence of multi-lineage structures. Mainstream approaches now include embryoid body (EB)-based suspension culture, co-assembly of pre-differentiated progenitors, and complex systems mimicking multi-germ layer interactions. These three primary strategies for generating 3D cardiac organoids are schematically summarized in Fig. 3 [36]. A systematic comparison of key recent protocols and the biological characteristics of the resulting cardiac organoids is presented in Table 1.

A representative protocol within EB suspension systems was established by Lewis-Israeli et al. [94]. It employs the GSK3 inhibitor CHIR99021 (4 µM, days 0–2) to activate Wnt signaling, followed by the PORCN inhibitor Wnt-C59 (2 µM) for timely inhibition, alongside low-dose BMP4 (0.36 pM) and Activin A (0.08 pM) for multi-stage regulation. This approach generated functional organoids comprising approximately 59% cardiomyocytes, 16% epicardial, and 14% endocardial cells, forming chamber-like structures with coordinated contraction and a preliminary vascular network within just seven days. To push organoids toward functional maturity, Volmert et al. developed a three-stage metabolic reprogramming system [95]. Their protocol employs a three-stage metabolic conditioning strategy to guide hiPSCs into forming electromechanically coupled, dual-chambered structures. The stages are: (1) a glycolysis-dependent phase, defined by culture in RPMI/B27 medium; (2) a fatty acid oxidation initiation phase, induced by the addition of L-carnitine/fatty acid-BSA complexes; and (3) a mitochondrial maturation phase, facilitated by supplementation with T3/IGF-1. Single-cell and spatial analyses showed that MYL7+ atrial-like and MYL2+ ventricular-like zones became spatially segregated, particularly under the EMM2/1 condition, consistent with improved chamber patterning. This model recapitulated the postnatal metabolic switch and established systematic criteria for assessing electrophysiological maturity. Another key innovation involves pre-differentiating hiPSCs into specific cardiac lineage progenitors prior to co-assembly. Hofbauer et al.‘s “cardioid” model exemplifies this approach [10]. Without non-cardiac tissues or exogenous ECM, it directs hiPSCs sequentially toward mesoderm, cardiac mesoderm, and spontaneously beating progenitors with > 90% efficiency via precise modulation of Activin, BMP, FGF, RA, and Wnt pathways, leading to rapid self-assembly into beating cavity structures. Building upon this progenitor co-assembly strategy, Schmidt et al. developed a multi-chamber platform that further elucidated pathway regulation in fate determination [11]. They demonstrated that stage-specific concentration gradients of Activin/Nodal, Wnt, BMP, and RA directly determine the fate of SHF, atrioventricular canal (AVC), and FHF progenitors. Timed inhibition of Activin/Nodal signaling was decisive for SHF commitment. RA signaling exhibited complex spatiotemporal functions: its stage-specific modulation contributed to anterior SHF induction, OFT differentiation, and chamber-specific lineage patterning.

To simulate more complex developmental crosstalk, multi-germ layer co-derivation models have been developed. Meier et al. constructed an epicardial-myocardial composite organoid by introducing 0.5 µM RA between days 2–5, successfully encapsulating a cTnT+ myocardial core with a WT1 + epicardial layer, thereby mimicking fetal left ventricular compaction [96]. Drakhlis et al.‘s cardiac organoids model, using Matrigel 3D culture, recapitulated the coordinated development of cardiac and foregut endoderm primordia, providing a unique platform for studying heart-endoderm interactions [97]. Venturing beyond traditional germ layer constraints, Silva et al. established a heart-gut co-development system [98]. Through dynamic Wnt regulation (switching from 12 µM CHIR99021 to 5 µM IWP2), they simultaneously generated functional myocardial and gut epithelial tissues within 3D agarose microwells, maintaining developmental synchrony for up to 100 days. The presence of endodermal tissues facilitated cardiac maturation features characteristic of the post-heart tube stage, including cardiomyocyte expansion, compartmentalization, and fetal-like functional maturation. Vascular integration is a crucial step toward physiological relevance. Ghosheh et al. generated multi-chambered, self-paced vascularized human cardiac organoids under anisotropic stress by co-culturing hiPSC-derived cardiomyocytes with rat primary cardiac microvascular endothelial cells [99]. A breakthrough came from Abilez et al., who cultivated highly vascularized cardiac organoids [100]. This model stably generated nearly all major human cardiac cell types and spontaneously forms spatially organized, branched, and lumenized vascular networks. Single-cell transcriptomics and high-resolution 3D imaging confirmed that vascularized cardiac organoids closely resemble the human fetal heart at approximately post-conception week 6.5.

These advances collectively mark a paradigm shift in cardiac organoid research, moving from simple structural mimicry to integrative systems biology modeling. The new generation of models, capable of recapitulating multi-lineage crosstalk, chamber specification, and vasculogenesis, provides an unprecedented platform for dissecting developmental mechanisms, modeling diseases, and performing high-fidelity drug screening.

**Table 1:** Summary of Cardiac Organoid Protocols and Corresponding Biological Outcomes

Author (Year)	Matrigel	Wnt modulation strategy	Key cytokines/growth factors	Media composition	Cellular composition	Morphological structure	Functional/developmental characteristics
Abilez et al. [100]	Yes	Activation: CHIR99021 (4–5 µM, 72 h) Inhibition: IWP (5 µM, 48 h)	FGF2, VEGF, SB431542, ANG1/2, PDGF-BB, TGF-β1	Essential 8 → RPMI 1640/B-27 (–insulin) → RPMI 1640/B-27	CM, EndoC, EpiC, EC, NC	Branched + lumenized vascular network; Multilineage structure	Recapitulates earliest cardiac + hepatic vascularization (first 3 weeks of human development)
Song et al. [103]	No	Activation: GSK3i (48 h) Inhibition: 48 h	BMP, VEGF, FGF, TGF-β	mTeSR1 → RPMI 1640/B-27 (–vitamin A) + 10 ng/ml TGF-β	51% CM, 25% FB, 14% EC	VE-cad⁺ cells on surface; Vim⁺ FB present	Higher expression of fibroblast + endothelial gene expression; predominantly MLC-2v⁺ ventricular-like CM
Dardano et al. (2024)	Yes	Activation: CHIR99021 (7.5 µM, 24 h) Inhibition: IWP (5 µM, 48 h)	BMP4, bFGF, VEGF, SCF, EPO, IL-6, IL-11, IGF-1, TPO, FLT3L, IL-3, SHH	Essential 8 → RPMI 1640/B-27 (–insulin) → RPMI 1640/B-27	CM, ST/proEpiC, arterial EC, venous EC, hemogenic EC, HPC, mature HC	Myocardial layer retained; Endoderm + EndoC-like layer lost; arterial/venous EC + hemogenic EC in outer layer; HC in outer layer	Recapitulates embryonic cardiac + haemato-vascular development
Schmidt et al. [11]	No	Activation: CHIR99021 (1–4 µM) Inhibition: Wnt-C59 (2 µM, 48 h)	Activin A, BMP4, FGF2, RA	Essential 8 → CDM (CDMI)	Progenitor subsets (FHF, aSHF, pSHF); Chamber-specified CM	Recapitulates all major embryonic heart compartments: LV, RV, atria, OFT, AVC	High similarity to early in vivo development
Meier et al. [96]	Yes	Activation: CHIR99021 (1.5 µM, 48 h) Inhibition: IWP2 (5 µM, 24 h × 4 cycles)	BMP4, Activin A, bFGF, VEGF	Essential 8 → DMEM/F12	CM, EpiC, MSC, EC	Dense CM core; thick EpiC layer; vessel-like structures	Recapitulates LV wall morphogenesis; molecular similarity to 6-week human embryonic ventricle
Volmert et al. [95]	No	Activation: CHIR99021 (4 µM, 24 h) Inhibition: Wnt-C59 (5 µM, 48 h) Secondary: CHIR (2 µM, 1 h, D7)	BMP4, Activin A, IGF-1	Essential 8 Flex → RPMI 1640/B-27 (–insulin) → RPMI 1640/B-27	aCM/vCM, VC, CC, EpiC, proEpiC, MSC	Two-chambered: atrial + ventricular chambers	Recapitulates post-heart tube stage development; high similarity to 6.5-week embryonic heart
Drakhlis et al. [97]	Yes	Activation: CHIR99021 (7.5 µM, 24 h) Inhibition: IWP (5 µM, 48 h)	Not specified	Essential 8 → RPMI 1640/B-27 (–insulin) → RPMI 1640/B-27	Myocardium (SHF-like), EndoC-like, serosa-like (WT1⁺), foregut endoderm (AFE/PFE), EC, liver progenitors	Myocardial layer lined with EndoC-like cells, surrounded by serosa-like cells; Foregut endoderm: IC (AFE) + OL (PFE/liver prog); Vascular network in IC	Recapitulates heart-foregut morphogenesis (pre-heart-tube stage)
Lewis-Israeli et al. [65]	No	Activation: CHIR99021 (4 µM, 24 h) Inhibition: Wnt-C59 (5 µM, 48 h) Secondary: CHIR (2 µM, 1 h, D7)	BMP4, Activin A	Essential 8 Flex → RPMI 1640/B-27 (–insulin) → RPMI 1640/B-27	59% CM, 16% EpiC, 14% EndoC, 12% FB, 1.6% EC	Multi-chamber + EndoC lining; vascular structures; EpiC layer adjacent to myocardium	Recapitulates fetal heart transcriptome
Silva et al. [98]	No (agarose wells)	Activation: CHIR99021 (12 µM, 24 h) Inhibition: IWP2 (5 µM, 48 h)	Not specified	mTeSR1 → RPMI 1640/B-27 (–insulin) → DMEM/F12 + L-ascorbic acid	CM, EndoC, EC, EpiC, SMC, gut epithelium	Multi-layer: EpiC layer + CM core + SMC layer adjacent to gut epithelium	Maturation to third-trimester fetal-like CM; atrial/nodal differentiation; spontaneous SMC peristalsis
Hofbauer et al. [10]	No	Activation (subtype-specific): CHIR99021 (1–9 µM, 36 h) Inhibition: IWP2 (5 µM, 96 h)	Activin A, BMP, FGF, RA	Essential 8 → DMEM/F12 → CDM (custom)	~ 90% CM	Single-chambered cavity	Exhibits early LV characteristics; organized sarcomeres + intercalated discs

Abbreviations: aCM, atrial cardiomyocytes; vCM, ventricular cardiomyocytes; CM, cardiomyocytes; CC, conductance cells; EndoC, endocardial cells; EC, endothelial cells; EpiC, epicardial cells; FB, fibroblasts; HC, hematopoietic cells; HPC, hematopoietic progenitor cells; MSC, mesenchymal cells; NC, neuronal cells; SMC, smooth muscle cells; ST, septum transversum; VC, valvular cells; Heart field identities: FHF, first heart field; SHF, second heart field; aSHF, anterior SHF; pSHF, posterior SHF; Foregut endoderm subregions: AFE, anterior foregut endoderm; PFE, posterior foregut endoderm; Structures: LV, left ventricle; RV, right ventricle; OFT, outflow tract; AVC, atrioventricular canal; IC, inner core; OL, outer layer; Others: RA, retinoic acid; FGF, fibroblast growth factor; VEGF, vascular endothelial growth factor; BMP, bone morphogenetic protein; TGF-β, transforming growth factor beta; CHIR, CHIR99021 (GSK3 inhibitor); IWP, inhibitor of Wnt production

## Applications of cardiac organoids

Cardiac organoids offer a highly biomimetic platform for modeling complex cardiac pathophysiological processes and hold particular promise for both congenital and acquired heart disease modeling. In myocardial infarction modeling, the study by Richards et al. recapitulated core features of acute myocardial infarction (MI) in 3D cardiac organoids by integrating physical oxygen gradients and chemical stress [101]. Using organoids with a radius of approximately 150 μm stimulated with 10% O₂ and 1 µM norepinephrine, they established an oxygen gradient mimicking human infarct zones: a central region where sharply decreased oxygen partial pressure triggered localized apoptosis, and a peripheral region maintaining viable myocardial characteristics. This synergistic effect of hypoxia and norepinephrine was crucial for inducing the pathological phenotype. Furthermore, the model responded favorably to the β-blocker metoprolol, underscoring its utility for drug intervention studies.

Also focusing on myocardial injury, Fernandes et al. employed combined stimulation with isoproterenol, hypoxia, and TGF-β1 to precisely mimic disease-associated metabolic and functional disturbances in mature cardiac organoids [102]. This model exhibited metabolic reprogramming, altered heart failure marker expression, and mitochondrial dysfunction. In contrast, these phenotypes were absent in immature organoids, highlighting the necessity of functional maturity for accurately modeling adult cardiac diseases. Song et al. modeled the continuum from acute myocardial infarction (AMI) to subsequent cardiac fibrosis [103]. By inducing stable hypoxia using cobalt chloride combined with glucose depletion and ischemia-reperfusion injury, they triggered characteristic AMI features in multi-lineage cardiac organoids. Subsequent TGF-β1 stimulation reproduced myocardial fibrosis, marked by aberrant fibroblast activation and extracellular matrix remodeling, providing a high-fidelity model for dissecting dynamic injury-repair interactions post-AMI.

In developmental heart disease modeling, a sustained high-glucose system constructed by Kostina et al. revealed molecular mechanisms underlying cardiac malformations induced by pregestational diabetes [104]. Exposure to 11.1 mM glucose induced cardiomyocyte hypertrophy and metabolic reprogramming compared to normoglycemic controls. Single-cell sequencing further revealed disrupted regulation of cardiac compartmentalization under high-glucose conditions. Collectively, these molecular changes closely mirrored complex cardiac malformation phenotypes observed clinically in fetuses from mothers with pregestational diabetes mellitus (PGDM), providing direct experimental evidence for understanding teratogenic mechanisms of metabolic disturbances.

Leveraging their 3D structure and multicellular composition, cardiac organoids show significant advantages in drug safety assessment, enabling accurate prediction of cardiotoxic responses. Mills et al. confirmed the platform’s value by functionally screening 105 small molecules with pro-regenerative potential [105]. This approach effectively circumvented false positives common in traditional 2D systems and successfully predicted drug effects on contractile function. Integrated high-content screening and proteomic analysis further revealed key pathways driving human cardiomyocyte proliferation. When evaluating known cardiotoxic drugs, the model accurately recapitulated typical doxorubicin-induced toxic responses, including release of cardiac injury markers, functional abnormalities, and mitochondrial dysfunction [106].

Research by Ghosheh et al. further validated cardiac organoids for evaluating detoxification strategies, showing that mitoxantrone-induced arrhythmias could be partially alleviated by metformin co-administration [99]. Schmidt et al. uncovered compartment-specific teratogenic mechanisms: thalidomide selectively inhibited atrioventricular canal development, retinoid-like drugs caused malformations via distinct mechanisms, and the pro-arrhythmic effect of Bay K 8644 was buffered in dual-chamber models due to electrical coupling [11]. Correspondingly, Volmert et al. confirmed dose-dependent ondansetron cardiotoxicity [95]. The induced ventricular wall thinning and electrophysiological abnormalities closely matched clinical risks of ventricular septal defects and fetal bradycardia, underscoring the role of organoids in early warning of medication safety during pregnancy.

In summary, these studies demonstrate that cardiac organoids provide a highly controllable and physiologically relevant platform for modeling complex cardiac diseases. Compared with traditional models, organoids offer improved fidelity in recapitulating multicellular interactions and dynamic pathological processes, thereby enhancing their value in both mechanistic studies and drug development.

## Challenges and outlook

By simulating the key molecular events of embryonic heart development, cardiac organoids formed based on hiPSC self-organization can recapitulate the important processes of early cardiogenesis, providing a high-fidelity research platform for cardiac disease modeling and drug toxicity assessment. Current research shows that self-organizing cardiac organoids have achieved a high degree of simulation of early human fetal heart development characteristics in terms of cell lineage completeness, successfully integrating multiple cardiac lineage cell types such as cardiomyocytes, endocardial cells, and epicardial cells [94, 95]. However, their functional maturity still faces significant challenges, mainly reflected in the following three aspects. (1) Electrophysiological immaturity: Electrophysiological maturation is a core dimension of cardiac function establishment, involving complex regulatory networks of ion channels and their subtypes within the cardiac syncytium. Studies show that the action potential upstroke velocity of organoid-derived ventricular-like cells under spontaneous conditions is generally below 10 mV/ms; even after hyperpolarizing the membrane potential close to adult levels via current injection (evoked conditions), the upstroke velocity is only around 20 mV/ms [97], far below the standard of 150–350 mV/ms for adult ventricular myocardium [107]. The resting membrane potential of adult ventricular myocytes is highly polarized (approximately − 90 mV) and lacks automaticity [108]; in contrast, organoid-derived cardiomyocytes currently exhibit an immature phenotype, with a maximum diastolic potential of only about − 70 mV [11]. Furthermore, the absence or underdevelopment of the transverse tubule (T-tubule) system is one of the hallmark features of organoid immaturity. Although some studies have observed tubular structures similar to T-tubules at the ultrastructural level, their characteristics remain similar to those of the early human fetal heart and lack the typical features of mature cardiomyocyte T-tubules [94, 109]. (2) Structural incompleteness: Current cardiac organoid models have not yet fully recapitulated the later stages and key processes of heart development, including valve formation, chamber septation, pacemaker region specification, trabeculation and ballooning, the establishment of the coronary vascular network and circulation, as well as overall heart growth and terminal maturation [11]. The absence of these structures limits the potential of organoids for modeling late-stage heart development and complex pathological conditions. (3) Metabolic immaturity: The human heart in its early developmental stage primarily relies on glycolysis for energy [110], while the transition to fatty acid oxidation is a critical step in late cardiac development [111]. Adult cardiomyocytes produce approximately 60–70% of their total ATP through fatty acid oxidation, whereas the metabolic profile of existing organoids remains skewed towards a fetal state: energy metabolism is predominantly glycolytic, mitochondria are underdeveloped, and oxidative phosphorylation levels are low [95].

In addition to self-organizing cardiac organoids, cutting-edge technologies such as tissue engineering, 3D bioprinting, and microfluidic organ-on-a-chip provide important complementary avenues for constructing cardiac models [112]. These technologies, through their respective unique advantages, synergistically advance organoid function towards human physiological characteristics: engineered human heart tissues, by integrating multiple cardiac lineages with biomimetic scaffolds, are dedicated to developing biological substitutes for structural myocardial regeneration [113, 114]; 3D bioprinting, relying on layer-by-layer precision deposition technology, achieves the spatial programming of cells and materials for complex cardiac geometries, with recent studies successfully constructing cardiac tissue containing embedded vascular networks, significantly enhancing the model’s mechanical stability and structural biomimicry [115]; microfluidic organ-on-a-chip technology utilizes miniaturized fluid control systems to dynamically simulate the cardiovascular physiological microenvironment, providing a highly biomimetic platform for high-throughput drug screening and disease mechanism studies [116, 117].

Particularly noteworthy is the deep integration of artificial intelligence with organoid technology, which is achieving intelligent optimization of construction strategies through algorithm-driven approaches. The application of AI is mainly reflected in five dimensions: efficient screening of construction strategies, low-cost extraction of image features, simplified analysis of multi-omics data, precise assessment for clinical translation, and systematic application development, significantly enhancing the physiological relevance, reproducibility, and standardization level of organoids [118].

## Conclusions

CVD has long been among the leading causes of death worldwide, and the study of its pathological mechanisms and the development of therapeutic strategies have long been limited by the inherent shortcomings of traditional models. The emergence of hiPSC technology has laid an important foundation for constructing human-specific cardiac models in vitro. The paradigm shift from culture systems relying on xenogeneic components to chemically defined, xeno-free culture systems has significantly improved experimental reproducibility and traceability, clearing a key obstacle for the differentiation of cardiac organoids and their clinical translational applications.

In summary, hiPSC-derived cardiac organoids—particularly advanced models integrating vascularized structures, multi-chamber morphology, and specific disease mutations—will continue to serve as a powerful platform, deepening our understanding of human heart development, disease mechanisms, and drug-heart interactions. Through continuously optimizing hiPSC culture systems and differentiation strategies, and deeply advancing interdisciplinary collaboration with fields such as tissue engineering, biomaterials, microfluidics, and artificial intelligence, cardiac organoid technology is expected to develop into a key bridge connecting basic scientific research with clinical diagnosis and treatment applications, providing innovative solutions for personalized medicine, regenerative medicine, and precision treatment of cardiovascular diseases.

## Data Availability

The data presented in this study are available from the corresponding author upon reasonable request.

## Abbreviations

AMI: Acute myocardial infarction
AVC: Atrioventricular canal
CVD: Cardiovascular disease
EB: Embryoid body
ECM: Extracellular matrix
FHF: First heart field
hESCs: Human embryonic stem cells
hiPSCs: Human induced pluripotent stem cells
hPSCs: Human pluripotent stem cells
MEFs: Mouse embryonic fibroblasts
OFT: Outflow tract
RA: Retinoic acid
SHF: Second heart f ield
